# A survey of American neurologists about brain death: understanding the conceptual basis and diagnostic tests for brain death

**DOI:** 10.1186/2110-5820-2-4

**Published:** 2012-02-17

**Authors:** Ari R Joffe, Natalie R Anton, Jonathan P Duff, Allan deCaen

**Affiliations:** 1 Stollery Children's Hospital and University of Alberta, Edmonton, Alberta, Canada; 2 The John Dossetor Health Ethics Center, University of Alberta, Edmonton, Alberta, Canada

## Abstract

**Background:**

Neurologists often diagnose brain death (BD) and explain BD to families in the intensive care unit. This study was designed to determine whether neurologists agree with the standard concept of death (irreversible loss of integrative unity of the organism) and understand the state of the brain when BD is diagnosed.

**Methods:**

A previously validated survey was mailed to a random sample of 500 board-certified neurologists in the United States. Main outcomes were: responses indicating the concept of death that BD fulfills and the empirical state of the brain that would rule out BD.

**Results:**

After the second mailing, 218 (44%) surveys were returned. Few (n = 52, 27%; 95% confidence interval (CI), 21%, 34%) responded that BD is death because the organism has lost integrative unity. The most common justification was a higher brain concept (n = 93, 48%; 95% CI, 41%, 55%), suggesting that irreversible loss of consciousness is death. Contrary to the recent President's Council on Bioethics, few (n = 22, 12%; 95% CI, 8%, 17%) responded that the irreversible lack of vital work of an organism is a concept of death that the BD criterion may satisfy. Many responded that certain brain functions remaining are not compatible with a diagnosis of BD, including EEG activity, evoked potential activity, and hypothalamic neuroendocrine function. Many also responded that brain blood flow and lack of brainstem destruction are not compatible with a diagnosis of BD.

**Conclusions:**

American neurologists do not have a consistent rationale for accepting BD as death, nor a clear understanding of diagnostic tests for BD.

## Background

There are two ways to diagnose death: irreversible loss of circulation, and irreversible loss of all functions of the brain, including the brainstem [1]. Each is a criterion for death, because it marks the univocal state of death, the irreversible loss of the function of the organism as a whole. Integrative unity of the organism, including resistance of entropy and maintenance of internal homeostasis, is lost, leaving a mere collection of tissues and organs [1-4]. For medicine, law, and ethics, this is the written standard rationale for accepting brain death (BD) as a criterion for death [1-4]. The tests used at the bedside to diagnose BD verify the irreversible loss of all functions of the brain. Neurologists in the intensive care unit confirm BD by using a clinical neurologic examination, and once diagnosed the patient is dead; this diagnosis is "final and cannot be reversed. The person will never awaken [5]." Some authors have challenged this paradigm [3,4,6]. In response, neurologist groups have made it clear that BD conforms with the law as written in the Uniform Determination of Death Act (UDDA), with "accepted medical standards" [7-11] and that "it will be hard to find a physician closely involved with BD determination and organ donation who does not think those [BD patients] are dead [9]."

We designed a survey to determine whether board-certified neurologists in the United States agree with the standard concept of death (defined by the President's Commission and neurologist groups as the irreversible loss of integrative unity of the organism [1-4,6,10,11]), and understand the criterion of death (irreversible loss of all functions of the brain, including the brainstem), and the empirical state of the brain diagnosed by the tests used to confirm BD. We hypothesized that neurologists would not be aware of the standard paradigm justifying the diagnosis of death and would not understand the empirical state of patients determined dead based on the criterion of BD. This is important because the American Academy of Neurology suggests that neurologists have special expertise in declarations of BD [7,8].

## Methods

### Questionnaire administration

This study was a prospective survey of a random sampling of board-certified neurologists in the United States regarding their opinions about BD. The mailing list was obtained from Healthcare Lists Division SDI (Yardley, PA) in August 2009. Each neurologist was mailed the survey in January 2010, along with a $5 gift certificate to encourage them to have a coffee while filling out the questionnaire. A cover letter asked participants to complete the survey and mail it back in the addressed, stamped envelope. A second mailing was done in May 2010 to nonresponders. All responses were received by July 2010. The cover letter stated, "We are sending you a short questionnaire asking your opinions around some of the concepts surrounding BD. We want to sample the opinions regarding the concept of BD. Your responses are voluntary and confidential." The study was approved by our university health ethics research board.

### Questionnaire development

The development and initial testing of the instrument are described in more detail elsewhere [12,13]. The current instrument (Additional File 1) is identical to that used in a survey of Canadian pediatric intensivists and Canadian neurosurgeons, with the following changes: (a) in the first question about acceptable conceptual reasons to explain BD, we added the choice "cessation of the vital work of a living organism--the work of self preservation, achieved through the organism's need driven commerce with the surrounding world" as stated by the President's Council; and (b) we modified the scenario regarding family refusal to stop "life support" in a brain-dead patient to describe continued support for 8 months until ventilator withdrawal, and asked "was this patient dead for the last 8 months?" and if the patient, during the last 8 months, was doing any of the three vital activities stated by the President's Council to indicate life (Additional File 1) [14].

To generate the items for inclusion in the questionnaire, we searched MEDLINE from 1996 to 2004 for articles on BD, followed by review of the relevant article reference lists. The new questions described above were based on the President's Council White Paper [14]. To ensure clarity, realism, validity, and ease of completion, initial pilot testing was done by having five local pediatric intensivists, one local pediatrician, and one local organ donation coordinator complete the questionnaire, followed by a semistructured interview for feedback.

### Statistical analysis

Certain definitions were made a priori for two of the survey questions. The first question asked the respondent to choose from a list of "stand-alone" reason(s) that "is/are an acceptable conceptual reason to explain why 'brain death' is equivalent to 'death'." The seventh question asked, "This patient fulfills all brain death criteria unequivocally, including the suitable interval. Conceptually, why are they dead (i.e., in your own words, what is it about loss of brain function, including the brainstem, that makes this patient dead)?" For analysis, we classified responses into categories that have been discussed in the literature, including loss of integration concept of BD, higher brain concept of BD, prognosis concept of BD, and statement of the criterion only.

Anonymous data were entered into REDCap Survey (Version 1.3.9-^©^2010 Vanderbilt University) and uploaded to the Statistical Package for the Social Sciences (SPSS, Inc., Chicago, IL) version 15.0 for Windows. We analyzed responses using standard descriptive tabulations and give adjusted Wald 95% confidence intervals (95% CI).

## Results

The questionnaire was mailed to a random sample of 500 board-certified neurologists in the United States; after the second mailing, 218 (44%) had been returned. Of the 218 returned, 26 (12%) did not have data that could be analyzed: 24 were returned to sender, and 2 were returned blank. Therefore, there were 192 of 477 (40.3%) eligible surveys returned with data for analysis.

The first question asked, "Which of several choices is an acceptable stand-alone conceptual reason to explain why BD is equivalent to death." Fifty-two (27%; 95% CI, 21-34%) chose the irreversible loss of the integration of body functions by the brain, 22 (12%; 8-17%) a cessation of the vital work of the organism, and almost half (48%; 41-55%) used a higher brain concept (Table 1).

**Table 1:** Responses to the question on conceptual reasons to explain why brain death is equivalent to death

Conceptual reason	Neurologist responses (n = 192)	95% Confidence interval
Higher brain concept	93 (48%)	41-55%

Irreversible loss of consciousness	82 (43%)	36-50%

Irreversible loss of the soul or "essence" of humans	39 (20%)	15-27%

Irreversible loss of "personhood"	43 (22%)	17-29%

Irreversible loss of the integration of body functions by the brain	52 (27%)	21-34%

Prognosis concept	59 (31%)	25-38%

The certainty of cardiac arrest within hours or days	14 (7%)	4-12%

Further care is futile and/or degrading	53 (28%)	22-34%

Restatement of loss of brain function (the criterion)	169 (88%)	83-92%

Irreversible loss of the function of the entire brain/brainstem	140 (73%)	66-79%

Irreversible loss of the critical functions of the entire brain/brainstem	105 (55%)	48-62%

Irreversible destruction of the brain, including the brainstem	109 (57%)	50-64%

Irreversible loss of the capacity for consciousness plus irreversible loss of the capacity to breathe	83 (43%)	36-50%

Cessation of the vital work of the organism	22 (12%)	8-17%

The exact question asked was as follows: "Which of the following is/are an acceptable *conceptual *reason to explain why 'brain death' is equivalent to 'death'?." Respondents could choose more than one answer; each answer had to be "a stand-alone reason." The standard medical, ethical, and legal conceptual reason is: the irreversible loss of the integration of body functions by the brain [1-4,10,11].

The next two questions asked about which objective test results, or pathology results (in a patient maintained as BD for 48 hours), would not be compatible with BD. A majority of respondents were unaware of the findings their patients may have when diagnosed with BD (Table 2).

**Table 2:** The objective findings that respondents considered would not be compatible with brain death

Finding	This would not be compatible with brain death (n = 192) [n (%; 95% confidence interval)]	**Actual percentage of clinically diagnosed brain death cases with this finding **[**15**,**16**]
**Objective test**		

Some EEG activity	135 (70%; 63-76%))	> 20%

Some evoked potential activity	107 (56%; 49-63%)	> 5%

Some cerebral blood flow	99 (52%; 45-59%)	> 5-40%

Some pituitary hormones	17 (9%; 6-14%)	> 50%

Normal brainstem pathology	36 (19%; 14-25%)	> 10-40%

None of the above	34 (18%; 13-24%)	Unknown

**Pathology finding**		

Brainstem minimal damage	81 (42%; 35-49%)	> 5-40%

Cerebral cortex minimal damage	63 (33%; 27-40%)	> 5-40%

Damage but not respirator brain	27 (14%; 10-20%)	> 5-40%

Widespread necrosis	1 (1%; 0-3%)	> 50%

None of the above	93 (48%; 41-55%)	Unknown

EEG = electroencephalogram

The standard medical, ethical, and legal tests for brain death only require clinical bedside tests; EEG, brainstem evoked potential, brain blood flow, or pituitary hormone testing are not required nor recommended [1,5,7,8,11,24,25]. In addition, brain pathology is not obtained as part of the diagnosis of brain death.

The next three questions asked about the timing of BD in different patient situations. When faced with a patient who has EEG activity yet fulfills BD criteria, 26 (14%; 9-19%) consider the patient dead at the first BD examination, 72 (38%; 31-45%) at the second examination, and 90 (47%; 40-54%) only when the EEG became isoelectric 12 hours later. When faced with a pregnant patient with BD supported for 11 weeks until delivery, most agreed the patient was dead by the first (36, 19%; 14-25%) or second (119, 62%; 55-69%) examination. However, in this brain-dead pregnant patient, 36 (19%; 14-25%) answered that she was not actually dead until sometime later: 11 (6%; 3-10%) after delivery of the neonate, 19 (10%; 6-15%) after organs are recovered and the ventilator is stopped, and 6 (3%; 1-7%) at none of these times. When faced with a brain-dead patient who has no cerebral blood flow but a family who insists on continued life support for the next months, and asked "was this patient dead for the last 8 months," 31 (16%; 12-22%) responded "no." When asked if this patient was performing vital work during those months, 164 (85%; 80-90%) responded no, and 30 (15%; 11-21%) responded yes [receptive to stimuli, 9 (5%; 2-9%); acting upon the world, 5 (3%; 1-6%), and carrying out basic (non-conscious) felt needs, 16 (8%; 5-13%)].

The next two questions asked again about the underlying conceptual basis of BD: "In your own words, what is it about loss of brain function including the brainstem that makes this patient dead?" and "Prior to this survey, had you thought about why, at a conceptual level, brain death is equivalent to death of the patient?" Only 21 (11%; 7-16%) of respondents had not previously thought about why BD is equivalent to death. In their own words, only 15 (8%; 5-13%) used a loss of integration concept (Table 3).

**Table 3:** Response to the question about what, in the respondent's own words, makes a patient dead

Concept given to justify why brain death is death	Neurologist responses (n = 192) [n (%; 95% confidence interval)]	Neurologists who agreed the conceptual basis makes brain death equivalent to death (n = 133) [n (%; 95% confidence interval)]
Higher brain concept	63 (33%; 27-40%)	52 (39%; 31-48%)

Loss of integration of body concept	15 (8%; 5-13%)	13 (10%; 6-16%)

Loss of integration alone	7 (4%; 2-7%)	7 (5%; 2-11%)

Loss of integration combined with higher brain concept	8 (4%; 2-8%)	6 (5%; 2-10%)

Prognosis concept	9 (5%; 2-9%)	5 (4%; 1-9%)

Prognosis of death certain	7 (4%; 2-7%)	3 (2%; 1-7%)

Quality of life statement	2 (1%; 0-4%)	2 (2%; 0-6%)

No concept given	96 (50%; 43-57%)	59 (44%; 36-53%)

Re-statement only: loss of brain function (the criterion)	32 (17%; 12-23%)	23 (17%; 12-25%)

No response (blank)	64 (33%; 27-40%)	36 (27%; 20-35%)

Vital work of organism concept^**a**^	4 (2%; 1-5%)	0 (0%; 0-2%)

Other	9 (5%; 2-9%)	4 (3%; 1-8%)

The exact question was as follows: "This patient fulfills all brain death criteria unequivocally including the suitable interval. *Conceptually*, why are they dead (i.e., in your own words, what is it about loss of brain function including the brainstem that makes this patient dead)?" The standard medical, ethical, and legal conceptual reason is (as defined by the President's Commission and neurologist groups): the irreversible loss of the integration of body functions by the brain [1-4,10,11].

^**a**^Responses were: "cannot independently sustain itself"; "irreversible loss of interaction with the environment and no ability to function"; "no longer capable of any activity that leads to self preservation"; and "the organism is no longer capable of interacting with the environment internally or externally."

The next question asked which choice "best describes why you are comfortable diagnosing death based on the criteria of brain death?" Most (133, 69%; 62-75%) responded that "the conceptual basis of brain death makes it equivalent to death of the patient." Many responded that the reason is because it is a standard: an accepted medical standard (46, 24%; 18-30%), an accepted legal standard (24, 13%; 8-18%), and/or "the diagnosis of brain death was taught to me during my training" (14, 7%; 4-12%). Five (3%; 1-6%) were not comfortable diagnosing death based on BD.

The final question asked: "Are brain death and cardiac death the same state (i.e., are both death of the patient)?" More than half (104, 54%; 47-61%) chose "no," 86 (45%; 38-52%) chose "yes," and 2 (1%; 0-4%) left the answer blank.

Further analysis was done for those 133 (69%) who responded that they were comfortable diagnosing BD, because "the conceptual basis of brain death makes it equivalent to death of the patient." Their responses to the question asking to state the concept of BD in their own words is shown in Table 3. Only 13 (10%; 6-16%) used a loss of integration concept, and 59 (44%; 36-53%) did not articulate a concept (i.e., used a restatement of the criterion or left no response). On the first question, only 39 (29%; 22-38%) considered "irreversible loss of the integration of body functions by the brain" as an acceptable conceptual reason to explain BD being equivalent to death and 67 (50%; 42-59%) chose a higher brain conceptual reason.

## Discussion

The American Academy of Neurology recently updated their evidence-based guideline for determining BD in adults, reaffirming that irreversible cessation of all functions of the entire brain, including the brainstem, can be determined "based on straightforward principles," and is death [8]. This survey suggests that there are several potential flaws with this claim. First, most neurologists do not understand (at best) or disagree (at worst) with the standard concept that BD is death because the organism has lost integrative unity. The most common justification given by neurologists was a higher brain concept, suggesting that irreversible loss of consciousness is death. Very few neurologists consider the irreversible lack of vital work of an organism as a concept of death that the BD criterion may satisfy. Second, most neurologists do not understand (at best) or disagree (at worst) that certain brain functions, including EEG activity, evoked potential activity, and hypothalamic neuroendocrine function, often can remain in patients diagnosed dead using accepted tests that have confirmed the BD criterion [15]. This suggests that these neurologists think that clinical tests for BD produce many false-positive diagnoses of death. Third, most neurologists did not understand (at best) or disagree (at worst) that brain blood flow and lack of brain destruction often can occur in patients diagnosed dead using accepted tests confirming the BD criterion [15,16]. This suggests that there may be concern (or confusion) about whether BD marks the point of irreversible loss of brain functions. Finally, most neurologists do not consider the criterion BD and circulatory death as each diagnostic of the univocal state of death.

### The concept of death

BD is said to be death by most professional bodies because it satisfies the concept/definition of death (Table 4): loss of integrative unity of the organism as a whole, marking when an organism is no longer an organism because it no longer can resist the forces of entropy and no longer can maintain internal homeostasis [1-4]. Many have argued that integrative unity of the organism as a whole often continues during BD (hence, integration is not dependent on functions of the brain), and a central integrator is not required for life; therefore, many no longer consider this a concept of death that BD satisfies [3,6,14]. Loss of personhood, based on irreversible loss of consciousness (sentience, or agency) is necessary, but not sufficient, for death (Table 4) [3,4]. Although nonconscious patients may be allowed to die due to their profound neurological disability, no society has accepted that they are already dead. It may be true that BD patients have poor quality of life or certainty of cardiac arrest in a short period; however, this denotes a prognosis and not a diagnosis of death. The President's Council suggested a novel concept of death: that vital external work of an organism is required to be alive, and once an organism no longer interacts with the environment to obtain what it needs to survive, it is dead [14]. Importantly, simply restating the criterion of BD does not give any concept of death that BD satisfies to justify BD being death.

**Table 4:** Conceptual and empirical arguments in favor of brain death, and problems with those arguments

The conceptual or empirical arguments in favor of brain death	Problems with the argument
**The concept of death fulfilled by the brain death criterion**	

Irreversible loss of integrative unity of the organism as a whole	Integrative unity continues during BD: there are many reports of gestation of a fetus, waste detoxification and excretion, assimilation of nutrients, fighting of infections, wound healing, proportionate growth, and sexual maturation [6,14]. Without intensive care, BD patients will surely die quickly; but this is similar to many intensive care patients who are clearly live integrated organisms, such as those with cervical spine injury, on extracorporeal life support, etc.
	
	A central integrator is not required: embryos are alive [3,17].

Irreversible loss of personhood, consciousness, or moral agency (higher brain)	Consciousness is not the dividing line between life and death: irreversible vegetative state, anencephaly, and if moral agency is required, infants and the severely demented are not considered already dead (appropriate for burial, cremation, autopsy, or organ recovery) [3,4,17].
	
	Although consciousness may be a sign of ongoing integration, it can be lost with continued integration of the organism as a whole [3,4,6,17].

Poor quality of life or certainty of cardiac arrest	Conflate prognosis of death with a diagnosis of death. A prognosis of lack of recovery of neurological function is not a diagnosis of death.

Irreversible loss of the vital external work of an organism interacting with the environment to obtain what it needs	Brain-dead bodies *are *receptive to stimuli/signals from the surrounding environment (e.g., clot blood at and heal tracheostomy and gastric tube incisions; have withdrawal spinal reflexes; react with hypertension and tachycardia to organ recovery).
	
	Brain dead bodies *do *act upon the world to obtain selectively what they need (e.g., assimilate nutrients/electrolytes from fluids/feeds; eliminate unneeded wastes in stool/urine; exchange gases with the world in ventilated lungs).
	
	Brain dead bodies *do *have basic (non-conscious) felt needs that drive the organism to obtain what it needs (e.g., the drive to circulate blood with oxygen/nutrients to sustain its vital organs, to absorb needed nutrients and eliminate unneeded wastes from the bowel, to acquire needed oxygen from the lungs) to allow growth, sexual maturation, and recovery from complications.
	
	The goal of external work is to sustain the "capacity for internal integrative unity": external work is "a second-order activity mandated by the primary work of an organism, the maintenance of internal homeostasis [19]."

Irreversible loss of the function (or the critical functions) of the entire brain, irreversible destruction of the brain, or irreversible loss of the capacity for consciousness and breathing.	These simply restate the criterion of brain death; they do not give a concept of death to justify the criterion being death itself.

**Empirical continuing brain activity after a valid clinical diagnosis of brain death is pronounced**	

Residual functions detected in brain death are actually mere activities (of "nests" of cells) and not functions.	The brain is too complex an organ to simply make this ad hoc and likely incorrect claim [3,17]:
	
	The spatial resolution of EEG suggests there is widespread neuronal activity when EEG activity is detected, potentially performing functions 317.
	
	Evoked potential activity is due to transduction of ambient energy into electrochemical signals conducted to the brain, suggestive of a function 317.
	
	Neuroendocrine control maintains free water homeostasis, suggestive of a function 34617.

Residual functions detected in brain death are insignificant functions.	This claim is ad hoc (without a clear reason): why are pupillary and corneal reflexes significant functions reflecting integration of the organism as a whole, while EEG activity, evoked potential activity, neuroendocrine control, and breathing at a PaCO_2 _of 80 mmHg are not [3,15,17]?

Residual functions are neither critical nor clinical functions, and BD is a clinical diagnosis.	This claim is ad hoc (without a clear reason): how to define critical, and why these must be clinical functions is not explained [3,15,17].
	
	The clinical versus nonclinical distinction is irrelevant: neurologists' epistemic access to a function is not a relevant consideration to diagnosis of a critical function [3,17].
	
	The clinical versus nonclinical distinction is false: neuroendocrine control can be diagnosed at the bedside by observing lack of polyuria [3,17].
	
	The critical versus noncritical distinction is circular: critical functions are necessary for maintenance of life, and death is the loss of critical functions, is a trivial tautologous argument [3,17].

Residual functions are not critical because they are replaceable mechanically.	Breathing can be replaced mechanically and, therefore, is not a critical brain function. Like the dialysis machine replacing spontaneous kidney function, the ventilator replacing spontaneous brainstem control of breathing is irrelevant as to whether an organism is dead [3,4,15,17,18].
	
	Only consciousness cannot be replaced mechanically and, therefore, this is only an argument for a consciousness based (not integration, or vital external work based) concept of death [3,4,15,17,18].

BD = brain death; EEG = electroencephalogram.

This survey shows that neurologists do not understand if, or disagree whether, the criterion BD fulfils a concept of death. Few consider irreversible loss of integration of the organism as a whole or irreversible loss of the ability to perform external vital work as a reason to accept BD as death (some even consider that external work continues during BD). Many confused a restatement of the criterion of BD as justification that it is death, and a few conflated the prognosis of death with the diagnosis of death. Most consider a higher brain concept of death justified. This is concerning because neurologists often are the specialist declaring BD and explaining it to families in the intensive care unit.

### The tests of BD

The tests for BD are performed to confirm that irreversible loss of all functions of the brain, including the brainstem, has occurred. It has been shown that some brain functions continue after accurately clinically diagnosed BD, including EEG activity in 20%, evoked potential activity in 5%, and hypothalamic neuroendocrine function in > 50% [15]. These activities may be explained by the finding that continued brain blood flow occurs in 5-40% of BD patients, and pathologic destruction of brain does not occur in more than 40% of BD patients (even after over 24-48 hr of maintained circulation) [15,16]. The ongoing brain functions have been explained with several controversial claims (Table 4) [3,4,15,17-19]. First, these are mere *activities *and not functions; however, the brain seems too complex an organ to simply make this claim [3,17]. Second, these are *insignificant *functions; however, this is an ad hoc claim [3,4,15,17]. Third, these are not critical *clinical *functions, and BD is a clinical diagnosis; however, this claim is both ad hoc and circular (critical clinical functions are necessary for maintenance of life, and death is the loss of critical clinical functions, is a trivial tautologous statement) [3,17]. Fourth, these are not critical functions, because they are *replaceable *mechanically; however, this would only lead to a higher brain consciousness based concept of death [3,4,15,17-19].

This survey shows that most neurologists do not understand, or disagree, that certain brain functions can remain in patients diagnosed dead using accepted clinical tests confirming the BD criterion. This may suggest that the accepted medical standard of clinical tests for BD can produce false-positive diagnoses of death. At the very least, the neurologists often are unaware of (or worse, disagree with) the debates regarding the meaning of significant, critical, clinical, brain functions.

### Other potential interpretations

First, we assumed that there is a "standard concept of death." However, we included in the survey all the concepts offered in the literature and also provided an opportunity to provide a new concept in the open-ended question. Although we found that most neurologists did not agree with the concept of loss of integrative unity, the main alternative was a higher brain concept. This would imply that patients with permanent vegetative state are dead in their state of wakefulness and breathing. Second, perhaps the finding that 97% of neurologists are comfortable diagnosing death based on BD only shows that neurologists are not able to justify explicitly why the equivalency truly holds. After all, this is a philosophical question and may not involve terminology used in clinical training. Perhaps the main finding of the survey is uncovering an unmet neurologists' educational need. Although a potential interpretation, this may not be reassuring to families who are told that their loved one is dead based on the criterion BD. In addition, the American Board of Psychiatry and Neurology lists an understanding of BD on the objectives of training [20]. Third, although the survey did not determine this, perhaps neurologists accept BD as "dead enough" for organ donation and withdrawal of life-support purposes. Accordingly, patients with BD should be allowed to die or should be treated as if they no longer are part of the human moral community; but, this is different than being biologically dead. We agree with other authors who have suggested that if BD is not death, whether BD can be considered a state where vital organ donation complies with nonmaleficence (death is an unavoidable and minimal harm) and autonomy (with informed consent) requires further discussion and debate [21].

### Limitations and strengths

The relatively small sample size, only modest response rate to this survey, and lack of information regarding respondents' exposure to BD patients are significant limitations. In addition, the closed-ended questions may not have allowed respondents to elaborate and clarify their responses. The strengths of the survey include the development methodology, and unambiguous nature of most of the questions. In addition, the striking similarity of our results to those of other surveys done in the past, including using this same survey in different populations of North American nonneurologist medical specialists, enhances the generalizability of the results [12,13,22-24]. The preponderance of evidence from this survey, and other surveys, support the conclusions we have drawn.

## Conclusions

Neurologists do not have a consistent rationale for accepting BD as death, nor a clear understanding of the diagnostic tests for BD. Almost half accept BD because it is a state of permanent unconsciousness, and more than half do not consider it equivalent to circulatory death. Wijdicks, in explaining that BD is a clinical diagnosis, and that confirmatory tests are not needed, asks "So, what are neurologists confirming?" [25]. Unfortunately, he does not answer this question, and only claims that "confirmatory tests do not confirm anything [because BD] is synonymous with a certain clinical state [from which] there are no recoveries on record.... [25]" Similarly, the American Academy of Neurology and the Canadian Forum Brain Death Guidelines suggest that BD is death because of its prognosis (claiming it is irreversible) and lack of consciousness [7,8,26]. If BD is death, a conceptual rationale for this should be clarified. This has important ethical implications for the practice of intensive care medicine.

## Competing interests

The authors declare that they have no competing interests.

## Authors' contributions

ARJ designed the study, analyzed the data, and drafted the manuscript. All authors (ARJ, NRA, JPD, ARD) contributed to study conception and design, acquisition of data, interpretation of data, revised the manuscript critically for important intellectual content, and have given final approval of the version to be published.

## Supplementary Material

Additional file 1 **Brain Death Survey**. The survey sent out to American neurologists asking for their opinions regarding brain death. Click here for file
