# Supplementary material for: A survey of American neurologists about brain death: understanding the conceptual basis and diagnostic tests for brain death
Source: Ann Intensive Care. 2012 Feb 17;2:4. doi: 10.1186/2110-5820-2-4 (PMC3310851; doi:10.1186/2110-5820-2-4)
Supplement: Additional file 1 — Brain Death Survey. The survey sent out to American neurologists asking for their opinions regarding brain death. [file 2110-5820-2-4-S1.DOC]

**A Survey of American Neurologists About Brain Death: Understanding of The Conceptual Basis and Diagnostic Tests For Brain Death**

**Journal Name:** Annals of Intensive Care

**Authors**: ^1,2^Ari R Joffe, ^1^Natalie R Anton, ^1^Jonathan P Duff, ^1^Allan R deCaen

**Affiliations:** ^1^Stollery Children’s Hospital and University of Alberta, Edmonton, Alberta, Canada; ^2^The John Dossetor Health Ethics Center, University of Alberta, Edmonton, Alberta, Canada.

**Corresponding author:** Ari R Joffe MD; Email: [ari.joffe@albertahealthservices.ca](mailto:ari.joffe@albertahealthservices.ca)

**Additional File 1: The survey instrument: survey of board-certified neurologists in the United States.**

All the following questions apply to a 16 year old patient after severe head trauma in a motor vehicle collision. ***The patient fulfils all clinical brain death criteria unequivocally including the suitable interval (in this case 12 hours)*.** In each question you may choose **any/all** of the responses that you consider correct. Please answer the questions on both sides of this page.

1. Which of the following is/are an acceptable *conceptual* reason to explain why ‘brain death’ is equivalent to ‘death’ (you may choose more than one answer; **each answer must be a stand alone reason**):

- irreversible loss of consciousness
- irreversible loss of the soul, or ‘the essence’ of man
- irreversible loss of ‘personhood’
- irreversible loss of the integration of body functions by the brain
- the certainty of cardiac arrest within hours or days
- irreversible destruction of the brain including the brainstem
- irreversible loss of the function of the entire brain, including the brainstem
- irreversible loss of the critical functions of the entire brain including the brainstem
- irreversible loss of the capacity for consciousness plus irreversible loss of the capacity to breathe
- further care is futile and/or degrading
- cessation of the vital work of a living organism- the work of self preservation, achieved through the organism’s need driven commerce with the surrounding world
- none of the above

1. Which of the following objective test results would **not** be compatible with brain death (i.e. would prevent you from releasing this patient for organ donation):

- some EEG activity that is not due to artifact.
- some visual, somatosensory, or auditory evoked potential activity that is not due to artifact.
- some cerebral blood flow on technetium flow study.
- normal levels of regulatory pituitary hormones including TSH, antidiuretic hormone, and others.
- normal appearance of brainstem grossly and microscopically (assuming that this could be done).
- none of the above

1. After brain death is pronounced, the body is maintained for 48 hours at family request to allow family members to arrive and visit, before organ donation. The clinical findings remain unchanged. After organ donation an autopsy is done. Which of the following would **not** be compatible with this patient having been brain dead (i.e. would have prevented you from releasing this patient for organ donation):

- The brainstem appears structurally normal, and microscopically has minimal damage.
- The cerebral cortex appears structurally normal, and microscopically has minimal damage.
- The brainstem and cerebral cortex are damaged; however, there are not widespread destructive changes typical of “respirator brain”.
- There is widespread damage and necrosis (liquefaction) of cerebral hemispheres and brainstem.
- None of the above

1. An EEG at the time of the second brain death exam shows some residual activity that is not artifact. Another 12 hours later the clinical exam still shows brain death unequivocally, and the EEG is now isoelectric. When was this patient brain dead (as opposed to dying):

- First brain death exam fulfilled.
- Second brain death exam fulfilled (12 hours after the first exam).
- At the time of the isoelectric EEG (12 hours after the second exam).
- None of the above

1. The patient is 22 weeks pregnant. A decision is made to continue ventilation and inotropes to support the fetus. After two weeks, the patient is off of inotropes, hemodynamically stable, and remains fully ventilated. 11 weeks later a healthy 33 week gestation neonate is delivered by cesarean section. She still fulfils all the criteria for brain death, and is released for organ donation. When was this patient dead?

- First brain death exam fulfilled.
- Second brain death exam fulfilled (in this case, 12 hours after the first exam).
- After delivery of the neonate.
- After organs are harvested and the ventilator is stopped.
- None of the above

1. The family cannot accept that the patient is dead with a beating heart, and insist on continued intensive care including ventilation Even on further discussion, and with a cerebral blood flow study confirming no flow, the family still insists on continuation of “life support”.

The patient is supported on a ventilator and with tube feeds for 8 months until ventilation was withdrawn and her heart stopped. During the 8 months the body assimilated nutrients and electrolytes from the fluids and feeds provided, eliminated unneeded wastes in stool and urine, circulated blood with oxygen and nutrients to its vital organs, clotted blood at and healed the tracheostomy and gastric tube incisions, and therefore grew, sexually matured, and recovered from pneumonia episodes.

1. Was this patient dead for the last 8 months?

 Yes

 No

1. Was this patient doing any of the following during the last 8 months?

 being receptive to stimuli and signals from the surrounding environment

 acting upon the world to obtain selectively what it needs

 having any basic (non-conscious) felt need that drives the organism to obtain what it needs

 none of the above

1. a) This patient fulfils all brain death criteria unequivocally including the suitable interval. *Conceptually*, why are they dead? (i.e. in your own words, what is it about loss of brain function including the brainstem that makes this patient dead?)

b) Prior to this survey, had you thought about why, at a conceptual level, brain death is equivalent to death of the patient?

- Yes
- No

1. Which of the following **best** describes why *you* are comfortable diagnosing death based on the criteria of brain death (**please only pick one answer for this question**):

- the conceptual basis of brain death makes it equivalent to death of the patient
- the diagnosis of brain death is an accepted medical standard
- the diagnosis of brain death is an accepted legal standard
- the diagnosis of brain death was taught to me during my training
- I have not thought about exactly why I accept brain death as equivalent to death.
- I am not comfortable diagnosing death based on the criteria of brain death

9. Are brain death and cardiac death the same state (i.e. are both death of the patient)?

- Yes
- No

Thank you very much for your participation in this survey.
